# Blueberries’ Impact on Insulin Resistance and Glucose Intolerance

**DOI:** 10.3390/antiox5040044

**Published:** 2016-11-29

**Authors:** April J. Stull

**Affiliations:** Department of Human Ecology, University of Maryland Eastern Shore, Princess Anne, MD 21853, USA; ajstull@umes.edu; Tel.: +1-410-651-6060

**Keywords:** blueberries, bilberries, strawberries, cranberries, berries, anthocyanins, diabetes, insulin, glucose, diabetes

## Abstract

Blueberries are a rich source of polyphenols, which include anthocyanin bioactive compounds. Epidemiological evidence indicates that incorporating blueberries into the diet may lower the risk of developing type 2 diabetes (T2DM). These findings are supported by pre-clinical and clinical studies that have shown improvements in insulin resistance (i.e., increased insulin sensitivity) after obese and insulin-resistant rodents or humans consumed blueberries. Insulin resistance was assessed by homeostatic model assessment-estimated insulin resistance (HOMA-IR), insulin tolerance tests, and hyperinsulinemic-euglycemic clamps. Additionally, the improvements in glucose tolerance after blueberry consumption were assessed by glucose tolerance tests. However, firm conclusions regarding the anti-diabetic effect of blueberries cannot be drawn due to the small number of existing clinical studies. Although the current evidence is promising, more long-term, randomized, and placebo-controlled trials are needed to establish the role of blueberries in preventing or delaying T2DM.

## 1. Introduction

Insulin resistance is a public health concern that can initially occur in the prediabetes stage many years before the diagnosis of type 2 diabetes mellitus (T2DM). Insulin resistance is defined as inefficient glucose uptake and utilization in peripheral tissues in response to insulin stimulation [1]. Insulin resistance in the prediabetes stage is a characteristic of glucose intolerance, which includes impaired fasting glucose (fasting plasma glucose (FPG) 100–125 mg/dL or 5.6–6.9 mmol/L) and/or impaired glucose tolerance (oral glucose tolerance test (OGTT) 2-h plasma glucose (PG) 140–199 mg/dL or 7.8–11.0 mmol/L) [2,3]. Prediabetes is a condition in which blood glucose levels are higher than normal, but not high enough to be classified as T2DM. The prediabetes stage is when corrective actions need to be implemented in order to prevent or delay the development of T2DM (FPG ≥ 126 mg/dL or ≥ 7.0 mmol/L; or OGTT 2-h PG ≥ 200 mg/dL or ≥ 11.1 mmol/L). Thirty-seven percent of adult Americans have prediabetes, which increases their risk of developing T2DM and cardiovascular disease [4]. To circumvent the health complications of T2DM and its related financial burdens, primary prevention before the disease actually occurs is warranted.

Lifestyle and dietary habits are major factors determining the development and progression of T2DM. Epidemiological studies reported that consumption of foods rich in anthocyanins, particularly from blueberries, were associated with a lower risk of T2DM and index of peripheral insulin resistance [5,6,7]. Blueberries belong to the genus *Vaccinium* and their health benefits may be attributable to the bioactive compounds, anthocyanins, which also have antioxidant properties [8,9,10]. Anthocyanins are polyphenols that belong to the flavonoid subgroup and they are the natural dark pigment color in plant foods [11]. The bioactive compounds are abundant in fruits and vegetables, such as berries, cherries, grapes, red onion, red radish, and purple potatoes [12].

Blueberries have become a popular fruit that gained the interest of the public and scientific communities due to their role in maintaining and improving health [13]. The scientific evidence supporting the anti-diabetic health benefits of blueberries is growing. Pre-clinical [14,15,16,17,18,19,20,21] and clinical [22,23,24,25] studies have found improvements in insulin resistance and glucose tolerance after blueberry consumption in obese and insulin-resistant rodents and humans. For many years, increased consumption of blueberries has been a folk remedy in Canada for treating T2DM [26]. This review will examine the effects of blueberries on insulin resistance and glucose intolerance, including evidence from dietary intervention studies that used rodents or humans with T2DM or at risk of developing the disease. An overview of mechanistic insights from cell culture and gut hormones will be explored. In addition, the review will also highlight the anti-diabetic effect of bilberries, which also belong to the genus *Vaccinium* and are known outside the United States as the “European blueberry”.

## 2. Anti-Diabetic Effect of Blueberries

### 2.1. Preclinical Dietary Interventions

**Figure 1:**
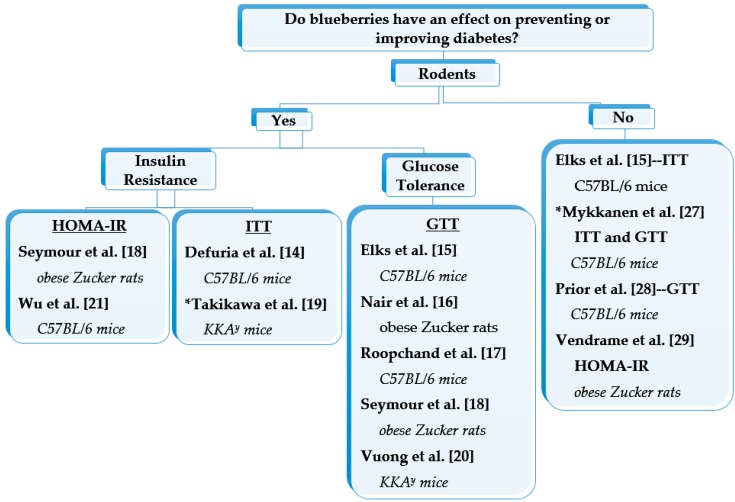
The effect of blueberries on preventing and improving type 2 diabetes in obese C57BL/6 mice, KKA^y^ mice, and Zucker rats. The rodents were fed blueberries for over 3 weeks and insulin resistance and/or glucose tolerance were assessed using HOMA-IR (homeostatic model assessment-estimated insulin resistance), ITT (insulin tolerance test), and GTT (glucose tolerance test). Seymour et al. [18], Mykkanen et al. [27], and Elks et al. [15] evaluated insulin resistance and glucose tolerance. * Studies that used bilberries.

Increasing insulin sensitivity (i.e., improving insulin resistance and glucose tolerance) is important in preventing or improving T2DM. A number of animal studies have demonstrated the anti-diabetic effects of blueberry anthocyanins (Figure 1). Obese rodents that were diet-induced and genetically manipulated consumed a 45%–60% high fat-diet (HFD) with blueberries for 3–12 weeks and their insulin resistance (i.e., assessed using the homeostatic model assessment-estimated insulin resistance (HOMA-IR)) [18,21] and glucose tolerance (assessed using the glucose tolerance test) [15,16,17,18,20] were improved. Similar results were observed when an intraperitoneal insulin tolerance test (ITT) was used to measure insulin sensitivity. DeFuria et al. [14] found that C57BL/6 mice that consumed a 60% HFD + 4% blueberries for 8 weeks had a lower plasma glucose area under the curve (AUC) (i.e., increased insulin sensitivity) during an ITT compared with the mice fed the HFD alone. The mice on the HFD + blueberries had similar results to the 10% low-fat diet fed mice. Additionally, similar increases in insulin sensitivity (assessed by ITT) were found in diabetic KKA^y^ mice that consumed a bilberry diet for 5 weeks [19].

In opposition to the previous positive anti-diabetic blueberry studies, other researchers documented no influence of blueberries on insulin resistance and/or glucose tolerance in obese mice and rats [15,27,28,29]. Although Vendrame and Colleagues [29] did not observe any significant changes on HOMA-IR with blueberry supplementation, they did find significant biological changes in the glucose metabolism related plasma markers (hemoglobin A1c, retinol-binding protein 4, and resistin concentrations). These markers were lower in the obese Zucker rats that consumed a 8% blueberry diet for 8 weeks when compared to the rats that did not consume blueberries. Also, the gene expression related to glucose metabolism (resistin in liver and retinol-binding protein 4 in adipose tissue) was downregulated in the obese Zucker rats following blueberry intake [29].

### 2.2. Clinical Dietary Interventions

#### 2.2.1. Whole Blueberries

**Figure 2:**
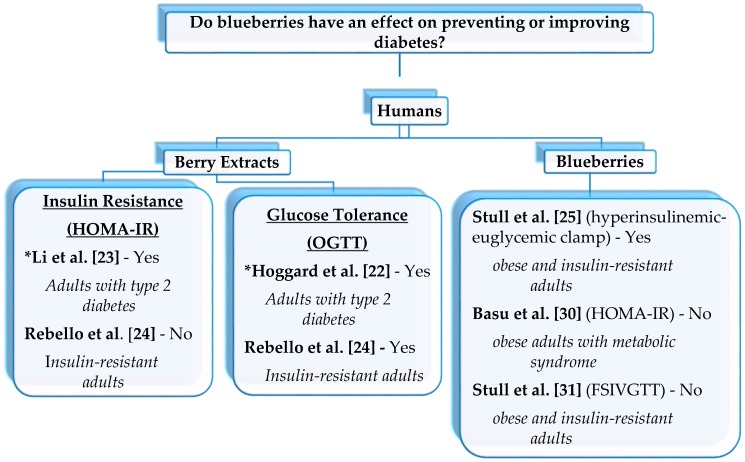
The effect of blueberries on preventing and improving type 2 diabetes in obese and insulin-resistant adults. Insulin resistance and/or glucose tolerance were assessed using HOMA-IR (homeostatic model assessment-estimated insulin resistance), FSIVGTT (Frequently sampled intravenous glucose tolerance test), and OGTT (oral glucose tolerance test). Rebello et al. [24], used HOMA-IR and OGTT and Stull et al. [25,31], used the clamp and FSIVGTT. * Studies that used bilberries.

In humans, evidence of blueberries impacting insulin resistance is sparse (Figure 2). Our lab group was the first to publish a report on the clinical impact of blueberries on whole-body insulin sensitivity in a population that was at risk for developing T2DM [10]. We found that consuming a smoothie supplemented with blueberries for 6 weeks had a greater increase in insulin sensitivity in obese and insulin-resistant adults (i.e., prediabetes) when compared to their counterparts that consumed a placebo smoothie. Insulin sensitivity was assessed by using the “gold standard” hyperinsulinemic-euglycemic clamp. Other studies, including our lab, have used less sensitive methods such as HOMA-IR [30] and frequently sampled intravenous glucose tolerance test (FSIVGTT) [31] to evaluate insulin sensitivity as a secondary measurement. Using these less sensitive methods resulted in no changes in insulin sensitivity between the blueberry and placebo groups.

#### 2.2.2. Blueberry or Bilberry Extracts

There are clinical studies that supplemented subjects with the blueberry or bilberry extracts instead of the whole berry (Figure 2). In overweight and obese subjects, Rebello et al. [24] used a gastrointestinal microbiome modulator (GIMM) containing inulin from agave, β-glucan from oats, and polyphenols from blueberry pomace as the dietary intervention. Consuming GIMM over 4 weeks improved glucose tolerance (assessed by oral glucose tolerance test; OGTT), but no changes in insulin resistance (assessed by HOMA-IR) were observed. Li and colleagues reported further evidence supporting the anti-diabetic role of berry extracts. The subjects with T2DM that consumed capsules containing 80 mg of anthocyanins (purified from the bilberry and blackcurrent; twice daily) for 24 weeks had a significant improvement in HOMA-IR (i.e., increased insulin sensitivity) [23]. Another study with a similar population distributed a single oral capsule of either 0 g (placebo) or 0.47 g standardized bilberry extract (36% *w*/*w* anthocyanins) to the subjects with T2DM [22]. This acute crossover design study found that supplementation with the bilberry extract resulted in a lower incremental plasma glucose and insulin (assessed by OGTT) when compared to consuming the placebo.

### 2.3. Ingredients in the Blueberry and Placebo Drinks, Pellets, or Capsules

The blueberry and placebo drinks, pellets, or capsules differed between the human and animal studies that were reviewed in Table 1. The reviewed studies varied in the types of berries, berry extract combinations, methods of administering the treatments (whole berry vs berry extracts), and contents in the food matrix. Potential concerns with the placebo that could influence the outcome data were not having a matched macronutrient placebo that was similar to the treatment and not controlling for fiber in the placebo. Thus, fiber has been shown to positively affect glucose control [32]. Another potential problem with the placebo is the added dark dye to make it indistinguishable from the treatment intervention. The chemical structures of the dark dyes are closely related to anthocyanins and this could possibly affect the study’s outcome variables. Regarding the food matrix, the smoothies in Stull et al.’s [25,31] study contained milk and yogurt and there is controversy about whether the proteins in milk interact with polyphenols and negate their antioxidant capacity and bioavailability. However, there are still discrepancies between studies [33,34,35,36,37]. It is important to note that the milk contained in the blueberry smoothie did not mask the beneficial effects of the blueberries on improving insulin sensitivity and endothelial function [25,31].

## 3. Prevention of Obesity-Potential Factor That May Contribute to the Anti-Diabetic Effect of Blueberries

The improved insulin sensitivity after blueberry supplementation that is exhibited in studies could possibly be due to the observed body weight and adiposity reduction in rodents. It is known that obesity is a major contributor to insulin resistance and changes in adiposity can greatly alter insulin sensitivity [38]. As seen in obesity, accumulation of lipids in tissues is a key step in the initiation and progression of insulin resistance to T2DM [38]. Increasing insulin sensitivity is important in preventing the development of T2DM. When blueberries are added to the diet, some studies have reported that obese rodents display a decrease in body weight gain and/or lipid accumulation in tissues with increased insulin sensitivity [17,20,21]. Contrarily, Prior et al. [28] observed increases in body weight gain and adiposity with blueberry consumption and this could possibly explain why blueberries did not influence insulin sensitivity in the obese mice. However, protection against obesity was observed when the obese mice were fed purified anthocyanins from blueberries [28,39]. Other researchers demonstrated increases in insulin sensitivity after blueberry consumption, but the blueberries were ineffective in reducing body weight gain and adiposity in the obese rodents [14,16,19]. Mykkanen et al. [27] observed the opposite and there were reductions in the body weight gain after bilberry supplementation in obese mice, but insulin sensitivity was not affected. In addition, Seymour et al. and colleagues [18] incorporated blueberries in the diet and the abdominal fat was reduced along with increases in insulin sensitivity in the obese Zucker rats. However, the total fat mass and body weight gain were unchanged during the 12 week study duration [18].

Body weight and fat composition have been mostly explored in animals, and to a lesser extent in humans. In clinical studies, body weight and fat composition have been explored as secondary measurements and the blueberry intake over 6–8 weeks did not change the body composition in the obese individuals [25,30,31]. Despite no changes in body weight and fat composition, Stull et al. [25] still observed an increase in insulin sensitivity after 6 weeks of blueberry consumption. Thus, it is possible that blueberries may help counteract obesity as seen in animal studies, but may not be as effective in inducing weight loss. Thus, clinical trials evaluating the anti-obesity effect of blueberries in humans is warranted with a longer study duration than 6–8 weeks.

## 4. Mechanisms of Action That are Related to the Anti-Diabetic Effect of Blueberries

### 4.1. Inhibition of Inflammatory Responses

Chronic inflammation is likely the link between obesity and insulin resistance [40,41]. Obesity is associated with macrophage infiltration into adipose tissue and the activation of the inflammatory pathway which leads to the development of insulin resistance [40,41]. The accumulation of macrophages in the adipocytes secrete proinflammatory cytokines. Previous animal studies have observed an anti-inflammatory effect of blueberries [14,27,42]. Vendrame et al. [42] reported that blueberries had an anti-inflammatory effect as evidence by a decreased expression in nuclear factor κB, interleukin-6 (IL-6), and tumor necrosis factor alpha (TNFα) in the liver and abdominal adipose tissue and decreased plasma concentrations in IL-6, TNFα, and c-reactive protein in obese Zucker rats. Insulin sensitivity was not evaluated in this particular study. Similarly, Defuria and colleagues [14] found that blueberries protected against adipocyte death, and down-regulation in gene expression indices of adipose tissue macrophage and inflammatory cytokines (TNFα and IL-10) in obese-induced mice. The researchers concluded that these changes in gene expression of inflammatory cytokines may have contributed to the increase in insulin sensitivity. Contrarily, an animal study reported increased insulin sensitivity, but no significant effect of blueberry intake on plasma inflammatory markers in obese Zucker rats [18].

In humans with hypercholesterolemia, consuming extracts from bilberry and blackcurrant anthocyanin significantly decreased the biomarker of inflammation on the vascular endothelium, soluble vascular cell adhesion molecule-1 (sVCAM-1), when compared to consuming the placebo [43]. When studies used the whole blueberry as the dietary intervention, the effects on the inflammatory response were less pronounced. Our research group [25,31] and other researchers [30,44,45] found that changes over 6–8 weeks in plasma levels of soluble intercellular adhesion molecule-1, sVCAM-1, C-reactive protein, IL-6, monocyte chemoattractant protein 1, and TNFα did not differ between the blueberry and placebo groups. Despite no changes in the inflammatory response, Stull el al. [25] still observed an increase in insulin sensitivity. Thus, in humans, a longer study duration, populations with elevated baseline inflammatory levels, and evaluation of the gene expression of the inflammatory markers may be necessary to observe an anti-inflammatory effect of blueberries on obesity and insulin resistance.

### 4.2. Modification of the Insulin-Dependent and Independent Cellular Pathways

There is evidence in both in vitro and in vivo models that suggest blueberries may modulate the intracellular pathways of glucose metabolism. However, there is still not a definitive answer for the cellular mechanism(s) that contribute to the anti-diabetic effect of blueberries. It is possible that there is more than one mechanism for blueberry-anthocyanins. Cell culture and animal studies have found that blueberry glucose uptake was due to activity in the insulin-dependent pathway [18,26] while other researchers have observed the activity in the insulin-independent pathway [19,46]. Contrarily, Roopchand et al. [17] found that blueberry-anthocyanins did not increase glucose uptake in L6 myotubes (i.e., skeletal muscle cells). However, these researchers did observe reduced glucose production in the H4IIE rat hepatocytes after adding blueberry anthocyanins.

Martineau et al. [26] showed that 21-h incubation of the blueberry (or fruit) extract in muscle cells enhanced glucose uptake only in the presence of insulin, which is an indication that the insulin-dependent pathway was utilized. Seymour et al. [18] reported that rats had an increase in mRNA transcripts related to glucose uptake and metabolism (e.g., insulin receptor substrate 1 (IRS 1) and glucose transporter 4 (GLUT 4)) in the skeletal muscle and retroperitoneal fat after consuming blueberries for 12 weeks. A different observation by Voung and Colleagues [46g] found the increase in glucose uptake was explained by the increased phosphorylation/activation of proteins in the insulin-independent pathway (e.g., AMP-activated protein kinase (AMPK)) in cultured muscle cells and adipocytes. Thus, the proteins in the insulin-dependent pathway (e.g., protein kinase B/AKT and extracellular signal-regulated kinase 1/2 (ERK)) were not affected by the blueberry treatments. In an in vivo study, bilberries activated AMPK in the white adipose tissue and skeletal muscle in KKA^y^ mice [19]. This activation induced upregulation of GLUT 4 and enhancement of glucose uptake and utilization in these tissues without using insulin. This data supported the previous evidence [46] that blueberries increased glucose uptake into the skeletal muscle cells and adipocytes via an insulin-independent mechanism. 

### 4.3. Other Mechanisms

Anthocyanins may have various anti-diabetic effects via mechanisms other than cellular signaling proteins found in the insulin-dependent and independent pathways, such as the modification of glucagon-like peptide-1 (GLP-1), alteration of peroxisome proliferator-activated receptor (PPAR) activities, protection against glucolipotoxicity, and modification of endogenous antioxidants. It is possible that anthocyanins can act directly within the intestine and exert health related benefits. Kato et al. [47] demonstrated that delphinidin 3-rutinoside (i.e., an anthocyanin) significantly increased GLP-1 secretion in GLUTag cells via the Ca^2+^/calmodulin-dependent kinase II pathway. GLP-1 is secreted from enteroendocrine L-cells and is one type of incretin that stimulates the glucose-dependent insulin secretion and proliferation of pancreatic β-cells. Increasing endogenous GLP-1 secretion is an alternative therapeutic approach that could possibly help treat diabetes and decrease the required medication doses [48,49,50]. The transcription factor PPAR was also observed and whole blueberries, isolated anthocyanins, and anthocyanin-rich extracts increased its activity [18,51,52,53]. PPARs are nuclear fatty acid receptors that play an important role in obesity-related metabolic diseases and PPAR agonist drugs have been used to improve insulin resistance [54]. In addition, the Chinese blueberry has been found to effectively protect β-cells against glucolipotoxicity when compared to metformin in vitro by reducing intracellular triglyceride levels, restoring intracellular insulin content, lowering basal insulin secretion, and increasing glucose-stimulated insulin secretion [55]. Glucolipotoxicity is a harmful effect of elevated glucose and fatty acid levels on pancreatic beta-cell function and survival [56]. Lastly, there are studies that demonstrated anthocyanins enhancing the endogenous antioxidant defense system. The purified anthocyanin cyanidin-3-*O*-β-glucoside increased glutathione (i.e., antioxidant) synthesis in the liver of diabetic db/db mice through upregulation of glutamate-cysteine ligase catalytic subunit expression [57]. Similar to the previous study [57], Roy and Colleagues [58] observed enhanced serum levels of superoxide dismutase and catalase after injections of pelargonidin (i.e., anthocyanidin) in the streptozotocin-induced diabetic rats.

## 5. Conclusions

Blueberries offer a natural “healthy package” of diverse bioactive compounds that contribute to its many health benefits. This review highlighted a multitude of in vivo and in vitro studies that demonstrated the anti-diabetic effects of blueberries and berry extracts in insulin-resistant rodent, human, and cell culture models. These beneficial effects of blueberries on insulin resistance and glucose tolerance in humans is in concordance with the animal and cell culture studies. Although there were studies that demonstrated a positive anti-diabetic effect of blueberries, this review also discussed studies with less pronounced effects. It is important to note that majority of the human studies that did not observe a positive outcome with whole blueberry supplementation used a less sensitive measurement to assess insulin sensitivity and also insulin sensitivity was a secondary measurement in the study.

The varying types of berries, berry extract combinations, the methods of administering the treatments (whole berry vs berry extracts), population studied, and the specifics of each study design bring a substantial amount of variation amongst the results in the various blueberry studies. There is a great need for more well designed, randomized, and placebo-controlled clinical trials that further explore dose responses, whole blueberries versus bioactive compounds, longevity of any health benefits, and interactions between blueberry bioactives and other foods and drugs. In addition, the cellular mechanisms are still controversial and findings are not consistent among studies. Therefore, more cellular mechanistic studies are warranted in in vivo models to elucidate the specific cellular signaling pathway involved in the improvement of insulin sensitivity after blueberry consumption. To date, there are a limited number of clinical studies that have evaluated the effect of blueberries on insulin sensitivity and more clinical trials are warranted before a definitive conclusion can be drawn about the anti-diabetic effect of blueberries.

## Figures and Tables

**Table 1:** Ingredients in the Blueberry Treatment and Placebo Drinks, Pellets, or Capsules.

	Study Type	BB Treatment	Placebo
**Blueberries**			
Lowbush (wild)			
*Vuong* et al. [20]	Pre-Clinical	BB juice (40 mL·kg^−1^ per day in drinking water)	water
*Prior* et al. [28]	Pre-Clinical	10% BB + LFD or HFD (pellets)	LFD or HFD (pellets)
*Vendrame* et al. [29]	Pre-Clinical	8% BB (pellets; regular diet)	pellets; regular diet
**Highbush**			
*Defuria* et al. [14]	Pre-Clinical	4% BB + HFD (pellets)	HFD (pellets)
*Elks* et al. [15]	Pre-Clinical	4% BB + HFD (pellets)	HFD (pellets)
*Roopchand* et al. [17]	Pre-Clinical	40% BB-defatted soyben flour (DSF) + HFD	HFD + DSF
*Seymour* et al. [18]	Pre-Clinical	2% BB + HFD (Semipurified diet)	HFD (Semipurified diet)
*Stull* et al. [25,31]	Clinical	22.5 g BB; 12 oz smoothie (yogurt and milk; 4 g Fiber) (twice daily)	12 oz smoothie (food color, BB flavor, and 4 g fiber) (twice daily)
*Basu* et al. [30]	Clinical	25g BB + 480 ml water (twice daily)	480 mL water (twice daily)
**Extract**			
*Rebello* et al. [24]	Clinical	BB ACN and polyphenols + 8.7 g fiber + 6 oz water (twice daily)	8.7 g fiber + 6 oz water (twice daily)
**Unknown (Highbush or Lowbush)**			
*Nair* et al. [16]	Pre-Clinical	2% BB + regular diet	corn + regular diet
*Wu* et al. [21]	Pre-Clinical	HFD + BB juice	HFD + water
**Bilberries**			
*Takikawa* et al. [19]	Pre-Clinical	27 g BB/kg + laboratory diet	laboratory diet
*Mykkanen* et al. [27]	Pre-Clinical	5% or 10% BB + HFD (pellets)	HFD (pellets)
*Hoggard* et al. [22]	Clinical	single gelatin capsule; 0.47 g of Mirtoselect^®^ (a standardized BB extract (36 % (w/w) of anthocyanins); ~50 g fresh BB	microcrystalline cellulose in an opaque single gelatin capsule
*Li* et al. [23]	Clinical	80 mg BB ACN + pullulan + maltodextrin capsule (twice daily)	pullulan + maltodextrin capsule (twice daily)

Abbreviations used: BB = blueberry or bilberry, HFD = 45% or 60% kcal high fat diet, ACN = anthocyanin.
